# The Moderating Role of Teamwork Engagement and Teambuilding on the Effect of Teamwork Competence as a Predictor of Innovation Behaviors among University Students

**DOI:** 10.3390/ijerph191912047

**Published:** 2022-09-23

**Authors:** Pilar Martín-Hernández, Marta Gil-Lacruz, Ana Cristina Tesán-Tesán, Amalia Raquel Pérez-Nebra, Juan Luis Azkue-Beteta, María Luz Rodrigo-Estevan

**Affiliations:** 1 Faculty of Social and Work Sciences, University of Zaragoza, 50009 Zaragoza, Spain; 2 Faculty of Health Sciences, University of Zaragoza, 50018 Zaragoza, Spain; 3 Social Capital and Wellbeing Research Group (B.Y.C.S.), University of Zaragoza, 22003 Huesca, Spain; 4 Faculty of Philosophy and Letters, University of Zaragoza, 50009 Zaragoza, Spain

**Keywords:** teamwork competence, innovation behaviors, teamwork engagement, teambuilding, higher education, sustainable development goals (SDGs), active learning methodologies

## Abstract

Sustainable innovation is the cornerstone of economic growth and development of regions and nations, as well as of organizational competitiveness and success. Innovation is a complex process that relies on individuals and often implies social activities based on interaction with others. Higher Education (HE) is expected to prepare innovative and teamwork-competent individuals. However, it has been noted that, so far, HE has not really addressed the question of how to train innovative college students capable of working in teams. To face such challenges, incorporating active teaching and learning methodologies, such as game-based learning (GBL), could be of great utility, as well as conducting much more research about the effect of teamwork-related factors on IWB, such as teamwork engagement (TWE) and teambuilding (TB). Under this background, our aim was to test the predictor effect of teamwork competence (TWE) on IWB, exploring the moderating role of TWE and TB in a sample comprising 142 college students of Health Sciences and Social Work. Our obtained results, through a multiple additive moderation analysis, showed that TWC positively predicted IWB. Moreover, this effect was moderated by TWE and TB. Therefore, these findings set light around how to foster IWB in HE.

## 1. Introduction

Innovation, “the creation and diffusion of new products, processes and methods” [1] (p. 18), is at the heart of economic growth and development of regions and nations [1,2,3], as well as of organizational success [4,5]. In fact, there is an innovation imperative: those organizations and nations that wish to flourish and survive need to introduce changes that imply an improvement in their products, services, and business models [6]. To meet and respond to market demands, organizations need to constantly develop and introduce innovations sustainably [7,8]. The Sustainable Development Goals (SDGs) [9], defined in Agenda 2030 for Sustainable Development, included innovation as a relevant goal (SDG9). Sustainable innovation must not only represent a competitive and business advantage for organizations. It must also give rise to environmental benefits and produce social welfare [10,11]. This social welfare must also reach the people who make up such organizations, favoring their development and health [5,12].

The innovative capacity of organizations resides in individuals. It is individuals, not organizations, who can identify and develop innovative ideas [13]. Individuals are the ones who can become most efficient in generating and introducing new ideas, frequently through collaboration and teamwork [14,15,16]. Therefore, individuals’ innovation work behaviors (IWB) are a key driving force behind organizational competitiveness and survival in the current globalized and complex business environment [17,18]. Thus, organizations are constantly searching not only for employees who add value to them through innovating, but also for human resource management (HRM) practices fostering IWB, such as job design, empowerment, teamwork, enhancing team trust and engagement [15,19,20,21,22,23,24,25,26]. This is particularly important. The urgent need for organizations to be innovative can make developing innovative performance an important demand for their workers, negatively influencing their well-being. By enhancing employee engagement as well as teamwork dynamics and competences through HRM practices, organizations are not only fostering their innovation capability, they are also contributing to the psychological and mental health of their employees. By doing so, such organizations are achieving another important sustainable development goal: SDG3 “Good Health and wellbeing” [9].

Strengthening IWB and favoring health and wellbeing, in accordance with the 2030 Agenda for Sustainable Development, are two important challenges not only for organizations but also for Higher Education institutions (HEIs). Today’s college students are tomorrow’s workers. HEI must prepare these students with suitable skills [27] to be able to insert themselves efficiently in businesses and the labor market. Actual business and the labor market need professionals who can generate and introduce innovations, as well as collaborate in innovation processes. Therefore, it is also the task of HEIs to prepare innovative individuals [28] capable of working in teams.

IWB is not only an emerging issue in HEIs [29] but also an unresolved one. On the one hand, as we will note, research has suggested that HEIs still have not really addressed the question of how to develop college students’ innovative capacity. On the other hand, the relationship between IWB and social interaction aspects in HEIs is still scarce explored. Therefore, little is known about the relationship amongst teamwork engagement (TWE) and other constructs, such as teambuilding (TB) and IWB in HEI. To face and solve such challenges, Higher Education (HE) must redouble its efforts, incorporating new and active teaching and learning methodologies, such as game-based learning (GBL), as well as developing more studies on how key aspects to work in teams, such as college students’ teamwork competences (TWC), TWE and TB influence IWB. Under this background, we examined the effect of such aspects related to teamwork on IWB in the context of a GBL experience in HE.

## 2. Theoretical Framework

### 2.1. Innovation Behaviors in HE

Innovation can be defined and conceptualized in several ways (see [28] for a wider review). In general terms, IWB usually involves the introduction, intentional and successful, of new ideas, processes and products [30]. In this regard, on the basis of the definition provided by West y Farr [31] (p. 9), IWB is conceived as “the intentional introduction and application within a role, group or organization of ideas, processes, products or procedures, new to the relevant unit of adoption, designed to significantly benefit the individual, the group, the organization or wider society”. IWB has been widely studied as a unidimensional construct [4] that comprises three distinct kinds of behavior, reflecting the three main stages of the innovation process [32], which are idea generation, idea promotion and idea realization [33]. Following Janssen [34,35], idea generation refers to the production of new ideas and is closely related to creativity; idea promotion involves finding help and support to carry out the new ideas; and, finally, idea realization implies the implementation of the newly generated ideas. In line with the more recent formulations, this conceptualization of IWB comprises individual creativity and remits to the importance of social aspects for IWB, such as discussing with others how to realize the new ideas. The production of new ideas can be an individual behavior, while IWB often implies an iterative and multistage process of production and implementation by team members in a group [15]. A recent study [15], conducted using a sample of 66 work teams with 275 vocational educators (teachers, school principals and social workers), showed the essential role of team reflexibility (e.g., continuous reflection on strategies and methods, processing knowledge and sharing it) for IWB [15].

In educational settings, including HEI, the study of IWB has been mainly focalized on the faculty and staff of such institutions, to a greater extent than on identifying what factors stimulated or inhibited innovation among college students. The still-scarce research conducted at this level has shown the effect of some individual factors (e.g., age, gender, prior levels of IWB, kind of college studies, information seeking behaviors, self-regulating learning, knowledge sharing behaviors, interpersonal competences) as well as organizational ones (e.g., course design characteristics, such as autonomy and cognitive demands [36]) and, to a lesser extent and more recently, social aspects, such as teamwork, team culture and communication [28,36,37,38,39,40]. Despite its value, this body of research is still far from providing a clear picture of which factors, especially those related to social interaction and teamwork, really matter regarding college students IWB in HEI.

In this vein, the distinctive role of HEIs in contributing to innovation as well as to the development of other key competences, such as teamwork, has been highlighted not only by research [41,42] but also by the renewed EU agenda for Higher Education institutions [43]. This renewed EU agenda also addressed the so-called “innovation gap” in HEIs, which is that the contribution of HEIs to innovation in the wider economy is often not as great as it should be. Therefore, HEIs must cope with the challenge of ensuring their sustainable contribution to innovation across all their activities, for example, training and equipping colleges students to think creatively and to develop and apply new ideas [41]. Engendering innovation often requires working in teams [15,16]. Team members must be able to generate new ideas and collaborate in translating them into new and improved working methods, procedures, products and services [18]. One promising avenue for HEIs to promote innovation and develop teamwork skills amongst college students in a healthy way, also allowing the achievement of SDGs, is to incorporate active learning methodologies (e.g., problem-based learning, gamification, game-based learning (GBL), flipped learning, learning by doing, service-learning (S-L), etc.) [44,45,46,47]. For instance, Martínez-Casanovas et al. [46] showed, in their recent study, that problem-based learning and case studies, such as active learning methodologies, promoted and enhanced SDGs.

Active learning methodologies place college students at the center of the teaching–learning processes, providing them opportunities for personal discovery, which improves the learning process [44], as well as for the development of their skills [48], such as creative thinking, problem solving, communication, teamwork and innovative performance [49]. In the last few decades, a growing body of research has shown the potential of these methodologies, such as GBL, to develop and foster amongst college students’ engagement, intrinsic motivation, academic performance, interaction, socialization, students’ confidence for teamwork and team building, creativity and innovation [50,51,52,53,54,55,56,57]. This body of research is undoubtedly of great value. However, a further step is needed on identifying factors that facilitate IWB in HEIs, especially those related with teamwork, such as teamwork competences and engagement. Few empirical studies have examined this issue to date in HEIs. In addition, a context in which active learning methodologies, such as GBL, have been applied, could provide an interesting opportunity to analyze how aspects related to teamwork have a significant impact, or not, on the innovative capacity of university students. Very briefly GBL experiences usually involve the playing a serious game in teams, combining inter-team competition and intra-team collaboration (e.g., cooperation, communication, sharing knowledge and ideas, team trust) to resolve and win it (see [49,58,59] for a longer description of GBL in HE). To achieve it efficiently and effectively, teams need their members to use their teamwork competences.

### 2.2. IWB and Teamwork-Related Factors

The study of IWB in HEIs has paid little attention to the social side in terms of teamwork competences, dynamics and outcomes, such as TWE. This might even seem surprising since both innovation and teamwork have long been pointed out in the literature as two key competences for the 21st century [60,61] that should be developed and strengthened in HEIs.

Teamwork competence (TWC) has been defined and conceptualized in different ways, both in workplace settings and HEIs. In general, it comprises a set of knowledges, skills and abilities (KSAs) [62] required to work effectively in a team [63]. This seemingly simple definition means, for example, that competent individuals in teamworking must be able to communicate properly to teammates and cooperate with them, contribute to carrying out the tasks entrusted to the group, both conceptual (e.g., researching and gathering information) and practical (e.g., providing constructive feedback) [64,65]. TWC plays a key role in innovation processes: it allows knowledge and ideas to be shared and combined, which, in turn, enhances innovative capabilities [27]. However, as we noted, empirical studies exploring TWC amongst college students as an antecedent of IWB are scarce in HEIs. A recent study based on 156 undergraduate students from a business school [63] found TWC positively predicted performance in an entrepreneurship education experience, in that creativity and innovation are essential [63,66]. The significantly positive association between TWC and IWB has also been found amongst workers across varied workplace settings, including educational ones [15,67,68,69]. Under this background, we hypothesize that:

**Hypothesis** **1** **(H1).** *TWC will positively predict IWB.*

This relationship between TWC and IWB can be further influenced by emergent states that reflect cognitive and affective properties and reactions (i.e., moods and thoughts) derived from team interaction [63], such as teamwork engagement (TWE) and team building (TB).

College students’ engagement—a positive individual state of mind characterized by energy, vigor, absorption and dedication [70,71]—is one of the most frequently reported outcomes derived from the use of active teaching and learning methodologies, such as GBL. Student engagement has been linked to self-esteem, satisfaction with studies and academic performance [62,72]. However, little research has examined this experience from a team point of view or, in other words, the so-called TWE. TWE is considered an emergent state “whose collective structure is shaped by the nature of their members’ interactions during team processes and dynamics” [73] (p. 34). TWE can be defined as “a shared, positive, fulfilling, motivational emergent state of work-related well-being” [73] (p. 35), characterized by teamwork vigor, dedication and absorption, positively associated with individual motivation and health, effectiveness and productivity [74]. Team members who experience higher feelings of TWE are more likely to be involved actively and efficiently in team processes and tasks, cooperating, assisting teammates and sharing knowledge and ideas. From this perspective, TWE can act as a catalyst by driving individuals to use their teamwork competences, fostering IWB. The effect of work engagement at the individual level, both direct and moderated, on IWB has been widely studied [69,75,76,77,78], mainly in workplace settings. However, as far as the authors know, present empirical research has not explored the moderating effect of TWE in the relationship between TWC and IWB in HEIs. Based on this, we formulate our hypothesis as follows:

**Hypothesis** **2** **(H2).** *TWE will positively moderate the relationship between TWC and IWB, strengthening it.*

The relationship between TWC and IWB in HEIs can be also moderated by TB. TB has been defined as “the formal and informal team-level interventions that focus on improving social relations and clarifying roles as well as solving task and interpersonal problems that affect team functioning” [79] (p. 9). In fact, it is conceived as a multidimensional construct that could entail interventions (e.g., using serious games [49,80]) that allow for the promotion of interpersonal relations, team trust, role clarification and the use of problem-solving and goal-setting techniques for the success of a project [79,81]. Thus, TB would capture team members’ perceptions of being and feeling as a team [79,82], properly organized and structured for the tasks that must be carried out [83]. As TB increases it, it could be expected that team members would be more likely to use their teamwork competences to innovate [84]. This relationship has hardly been studied; therefore, we propose our hypothesis in this regard in an exploratory way,

**Hypothesis** **3** **(H3).** *TB will moderate the relationship between TWC and IWB.*

Considering the evidence presented above, analyzing separately in the same model, the moderating effects of TWE and TB in the TWC–IWB relationship in the context of a GBL experience in HEIs could be useful for broadening the knowledge regarding the underlying mechanisms in such a relationship. It would make it also possible to establish more precise curricula guidelines to prepare innovative individuals capable of working in teams, contributing, in doing so, to the sustainability of innovation. Thus, the main objectives of this study are threefold. First, we test the predictive effect of TWC on college students’ IWB. Second, we aim to determine whether TWE positively moderates the relationship between TWC and IWB, acting as a catalyst for the effect of TWC on IWB. Finally, we examine whether TB moderates the positive effect of TWC on IWB.

**Figure 1:**
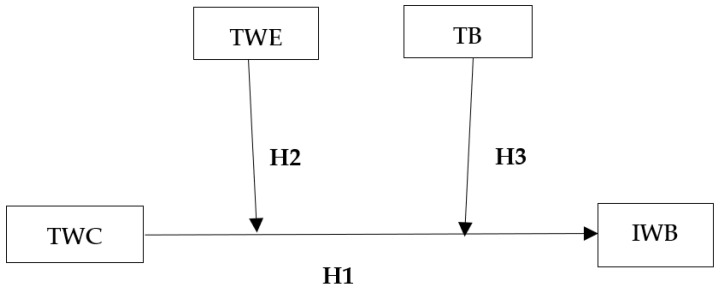
Conceptual model and proposed hypotheses.

Figure 1 illustrates our conceptual model and proposed hypotheses.

## 3. Materials and Methods

### 3.1. Participants

The convenience sample in this study was made up of 142 Occupational Therapy, Physiotherapy, Psychology and Social Work college students at the University of Zaragoza (Spain) (8.5%, 32.4%, 38% and 21.1%, respectively, of the whole sample). To establish the power of the analysis, we used G*Power 3.1.9.7 tool [85,86]. For linear multiple regression (fixed model, six predictors) the estimated sample size for this study (with an effect size of 0.35, a theoretical statistical power of 0.95 and an α = 0.01) is 86 participants. The main inclusion criteria of the research sample were that all of them, as a part of their university training, were enrolled in subjects related to the psychology of groups and participated voluntarily in the study. Further, 18.2% were in the first year of the degree, 44.1% in the second and 37.8% in the third. The age range of participants was 18 to 25 years old (M = 20.58; SD = 1.61). Further, 72.5% of the total sample were women.

### 3.2. Procedure

This cross-sectional study is part of a larger study interested in the effect of GBL on various aspects, including teamwork competences, dynamics and outcomes, as well as IWB in HEIs. That larger research had a longitudinal design with two data collections (T1 and T2), from February until the first week of March 2020. More concretely it consisted of the application of an experience of GBL using a serious game named “The group to the rescue” [87,88]. This serious game was based on theoretical and empirical foundations of the psychology of groups regarding the so-called effects of the physical, social and personal environment on group dynamics and performance. It was played by college students in small groups of five people interacting face to face in the classroom (see for wider description [49]). Participants completed, before (T1) and after (T2) playing the game, the anonymous pen-and-paper instrument used to measure the researched variables with a unique subject identifier (ID). All of them were informed that all participation was voluntary without any compensation, as well as of the objectives and anonymity of data collected. These statements were also described in the informed consent that they all accepted. The study was approved by the Research Ethics Committee of the Autonomous Community of Aragon (C.I. PI19/446).

The research conducted by Martín-Hernández et al. [49,87,88] provided support for the enhancing of IWB, TWC, TWE and TB through GBL, in line with all the empirical evidence that we discussed in the introduction [50,51,52,53,54,55,56,57]. However, we note also a further step is needed on identifying factors that facilitate IWB in HEIs, especially those related with teamwork, such as TWC, TWE and TB. Therefore, in this study we use the measurements in T2 for all these variables, except in the case of IWB since we will also take the measurement in T1 to consider those previous levels of IWB amongst colleges students in our analyses as a control variable.

### 3.3. Instruments

#### 3.3.1. Innovation Behaviors (IWBs)

Janssen’s nine-item instrument [34,35] was used to measure self-rated innovative performance by students themselves. Three of these nine items referred to idea generation (e.g., generating original solutions to problems), three to idea realization (e.g., introducing innovative ideas in a systematic way) and the remaining three to idea promotion (e.g., acquiring approval for innovative ideas). A 7-point scale ranging from never (1) to always (7) was used to code responses. Previous studies found intercorrelations among this three IWB components above 0.79 [32], as well as good results in terms of the scale validity in our sample [49]. Therefore, to obtain a global IWB score, we combined nine item scores. Higher scores indicated higher levels of innovative behaviors. The Cronbach’s alpha was 0.95 [49].

#### 3.3.2. Teamwork Competence (TWC)

Students’ perceptions regarding their own sense of competence for teamworking were measured using an adapted version of the instrument effectiveness in teams [64,65]. This 21-item instrument asked college students for their own teamwork behaviors related to team member effectiveness as conceptual and practical contribution to the teamwork (e.g., researching and gathering information), cooperation (e.g., assisting teammates when needed) and working ethically within the team (e.g., respecting commitments). All items were ranged on a 6-point Likert scale from 1 (completely disagree) to 6 (completely agree). The validity of this scale in our sample was test by Martín-Hernández, et al. (2021) [49] who returned good results [88]. To obtain a global-scale punctuation, we summed and averaged all item scores, with higher scores indicating higher levels of confidence in teamwork competence. Cronbach’s alpha was 0.92 [49].

#### 3.3.3. Teamwork Engagement (TWE)

Students’ feelings of teamwork engagement were assessed using the Spanish version [89] of the Utrecht Work Engagement Scale (UWES) [70] adapted to teams. This instrument consisted of 18 items and measuring three subscales: absorption (e.g., “We get carried away with work”); dedication (e.g., “We enjoy doing the work”) and vigor (e.g., “We feel strong and energetic during work”). Respondents answered using a 7-point Likert scale ranging from 1 (Never) to 7 (Always). In previous studies [83,90,91], this TWE scale presented good factorial validity, internal consistency and discriminant validity as well as in the one conducted by Martín-Hernández et al. [49] in the sample of the present study. Therefore, the subscales were combined to create a total engagement score, with higher scores indicating higher TWE feelings. Cronbach’s α for this scale was 0.95 [49].

#### 3.3.4. Team Building (TB)

College students’ experience of team building was measured using a 12-item instrument elaborated based on previous studies [92,93,94]. This scale allowed us to capture individuals’ perceptions in regarding their own sense of being a team inside his or her work group (e.g., “The group is organized and structured appropriately for the tasks that has to perform”). All items were ranged on a 6-point Likert scale from 1 (completely disagree) to 6 (completely agree). Martín-Hernández, et al. (2021) [49] tested the validity of this scale in our sample and returned good results. To obtain global-punctuation item scores, scores were summed and combined, with higher scores indicating higher TB experience. Cronbach’s alpha was 0.93 [49].

#### 3.3.5. Control Variables

Participants reported gender (0 = male, 1 = female) and age (in years). We controlled for these factors frequently considered as control variables in earlier IWB studies. Whereas research did not show consistent findings at this level reporting non-significant [36] as well as significant results, males as well as older individuals [29,37] were found to be more innovative. Furthermore, we also controlled for prior levels of IWB in T1 (that is, before playing the serious game “The group to the rescue”) measured using Janssen’s nine-item instrument [34,35]. The Cronbach’s alpha was 0.92 [49].

### 3.4. Data Analyses

SPSS 26.0 (IBM^®^, Armonk, NY, USA) and macro-PROCESS v. 4 [95,96] that allows for multiple moderation analysis (Model 2) to be conducted in SPSS were the software tools used for the data analyses. We used model 2 in that it allows one to estimate the effects of several moderators separately in the same model, showing the relative relevance of each moderator in the relationships under investigation. We included descriptive and internal consistency analyses (Cronbach’s alpha) and Pearson correlations for all variables in the Results section. Preliminary differential analyses and moderation model were conducted after checking assumptions to ensure their application (e.g., normality *p* > 0.05 (Kolmogorov test), homoscedasticity *p* > 0.05 (Levene test) and independency of the variables D-W = 1.83 (Durbin-Watson)). To estimate potential differences among the four degree groups of students (i.e., Physiotherapy, Psychology, Occupational Therapy and Social Work) in the variables analyzed measured after playing the game, we carried out a one-factor ANOVA with fixed effects (using if needed it post hoc Dunn test with Bonferroni adjustment). Model 2 was applied according to Hayes [89] to test for the moderation relationships, with a 95% CI and a bootstrapping of 10,000. *p* values < 0.05 and LLCI and ULCI were included in the model to support the significant effect of the variables in the model. IWB was entered into the model as a dependent variable, TWC as a predictor variable and TWE and TB as moderators. Following Hayes’ recommendations, the investigation of moderation was performed even when the predictors did not show significant associations with the DV in the previous regressions. Potential main effects of control variables (i.e., gender, recoded as a dummy variable (men = −0.5, women = 0.5) and age) as well as individuals’ IWB prior levels were introduced as covariates.

## 4. Results

Firstly, we offered the distribution of our sample by gender and age. Second, we conducted a one-way ANOVA to test potential differences in means of IWB, TWC, TWE and TB among the four groups of students participating in the study. Third, we performed descriptive, consistency and correlational analyses. Finally, we tested the role of TWC, TWE and TB in the prediction of IWB.

### 4.1. Descriptive, Correlacional and Internal Consistency Analyses

Table 1 shows the distribution by gender in each degree group.

Table 2 displays the results of the one-way ANOVA conducted to test potential differences in means of IWB, TWC, TWE and TB among the four groups of students participating in the study.

As shown in Table 2, no significant differences emerged (F = 2.31, *p* > 0.079; F = 1.83, *p* > 0.144; F = 1.89, *p* > 0.145 and F = 2.24, *p* > 0.086, respectively).

### 4.2. Correlational and Internal Consistency Analyses

Table 3 displays the results of descriptive, correlational (Pearson) and internal consistency analyses.

As shown in Table 3, IWB was positively associated with all the variables considered, except college student age and gender. Significant correlations varied from low to high, TWC being the strongest influence on IWB. In sum, the higher TWC (r = 0.72, *p* < 0.01), TWE (r = 0.57, *p* < 0.01) and TB (r = 0.55, *p* < 0.01), the higher the innovation behavior levels exhibited by the university students.

### 4.3. Multiple Additive Moderation Analysis

Table 4 displays the obtained results from the multiple additive moderation analysis conducted (Model 2 [89]).

**Figure 2:**
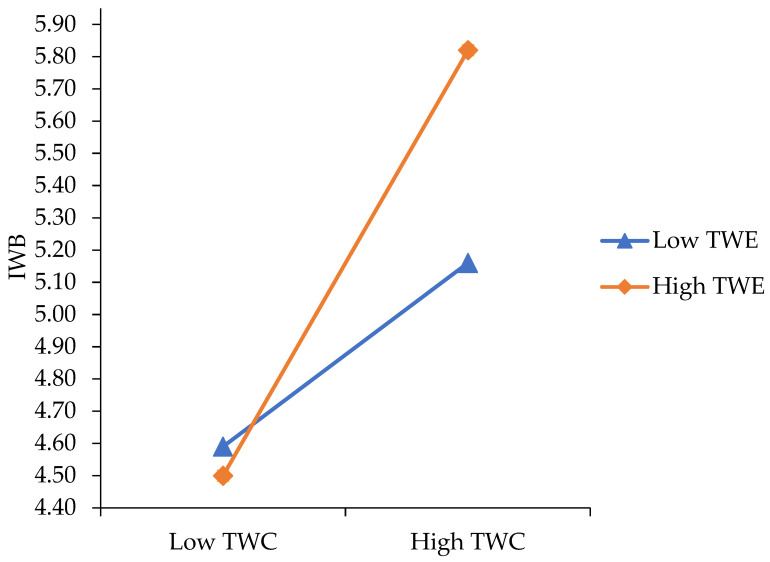
Relationship between TWC and IWB at low and high levels of TWE.

**Figure 3:**
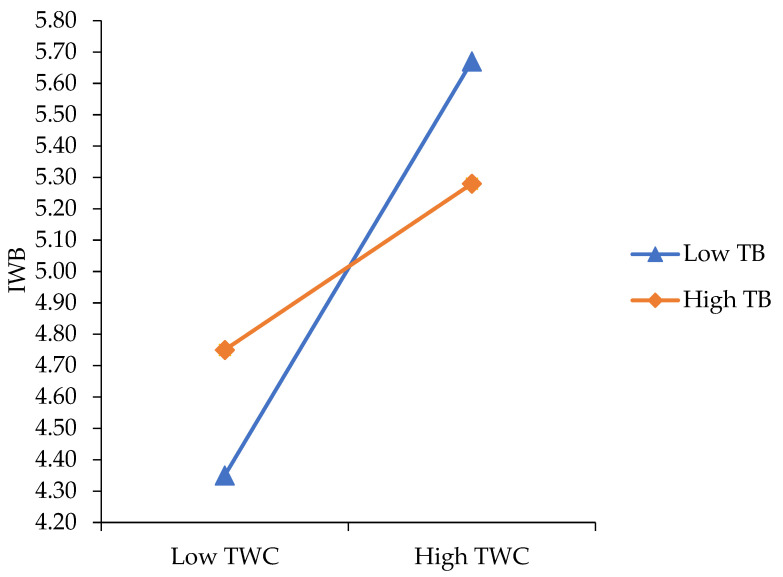
Relationship between TWC and IWB at low and high levels of TB.

The model was statistically significant (R^2^ = 64%, F = 40.41, *p* = 0.00). After controlling for age, gender and prior levels of IWB, TWC (β = 0.78, *SE* = 0.13, *t* = 6.10, 95% CI (0.53, 1.04), *p* = 0.00) positively predicted IWB. TWE and TB did not exert a significant direct effect on IWB. Obtained results supported a two-way interaction in the prediction of IWB between TWC and TWE (β = 0.37, *SE* = 0.12, *t* = 3.01, 95% CI (0.13, 0.61), *p* = 0.00), accounting for 2% of the variance in IWB (F _(1, 133)_ = 9.06, *p* < 0.001), as well as between TWC and TB (β = −0.46, *SE* = 0.15, *t* = −3.03, 95% CI (−0.76, −0.16), *p* = 0.00), accounting for 2% of the variance in IWB (F _(1, 133_) = 9.19, *p* = 0.00). Thus, H2 and H3 were supported. To interpret these interactions, we plotted separate regression slopes at one standard deviation above and below the mean, as shown in Figure 2 and Figure 3, respectively.

We found that TWE moderated the effect of TWC on IWB (β = 0.37, *SE* = 0.12, *t* = 3.01, 95% CI (0.13, 0.61), *p* = 0.00). As we noted, to interpret this interaction, we plotted separate regression slopes at two levels of TWE: low (i.e., 1 *SD* below the mean) and high (i.e., 1 *SD* above the mean) in Figure 2.

At low levels of TWE, IWB increased as college students developed higher levels of TWC. At high levels of TWE, we found a similar pattern. These results indicated that TWC is more strongly associated with IWB when TWE is high.

We also found that the effect of TWC on IWB was moderated by TB (β = −0.46, *SE* = 0.15, *t* = −3.03, 95% CI (−0.76, −0.16), *p* = 0.00). To interpret the interaction, we plotted separate regression slopes at two levels of TB: low (i.e., 1 *SD* below the mean) and high (i.e., 1 *SD* above the mean) in Figure 2.

At low levels of TB, IWB decreased as TWC was lower, whereas those college students with higher TWC developed more levels of IWB. At high levels of TB, the higher the TWC, the higher the levels of IWB exhibited by college students.

## 5. Discussion

This study analyzed the predictive effect of TWC on colleges students’ IWB, examining whether the relationship between TWC and IWB was further influenced by two important emergent states reflecting cognitive and affective properties and reactions derived from team interaction [63], such as TWE and TB. To achieve this goal, we used a sample composed by 142 Occupational Therapy, Physiotherapy, Psychology and Social Work college students at the University of Zaragoza (Spain), testing our hypotheses in a context in which an active learning methodology, such as GBL, was applied and played in small teams.

The first point of interest in our findings is that in the previous analyses conducted, no significant differences were found in the levels of IWB amongst the four groups of college students participating in the study. As Martín-Hernández et al. [49] noted, this could be due to the similarities in the training received in their degrees in Health Sciences and Social Work. Gender and age of the participants did not represent significant influences regarding IWB, in line with research that also showed non-significant relationships between these variables [36]. We also found that prior levels of IWB significantly influenced subsequent levels of innovation among college students in a positive way [36].

Our results supported all the formulated hypotheses (H1, H2 and H3). Teamwork competence (TWC) strongly and positively predicted IWB (H1), in accordance with previous studies, mainly conducted amongst workers across varied workplace settings, including educational ones [15,67,68]. This finding can be added to that little empirical research that analyzed the effect of TWE on IWB in HE [63,66]. As we commented in the Introduction section, despite the generation of new ideas often being an individual behavior, IWB often implies an iterative and multistage process of production and implementation by team members in a group [15,16]. Those college students who are more competent in teamwork would be better able to communicate and cooperate adequately with their teammates and to contribute, both from a conceptual and practical point of view to the accomplishment of the tasks entrusted to the group [64,65]. This greater competence for teamwork would allow them to share and combine knowledge and ideas to a greater extent and, therefore, to be more innovative [27].

Our results showed that the positive and significant effect of TWC on IWB was further influenced by two important emergent states derived from team interaction [63], such as TWE and TB, supporting H2 and H3, respectively. The multiple additive moderation analysis moderated the relationship between TWC and IWB, accounting for 2% of the explained variance in IWB. Regarding the moderating effect of TWE, we found that those college students with higher TWC who experienced higher levels of TWE were more innovative than those who experienced lower levels of TWE. Thus, TWC is more strongly associated with IWB when TWE is high. This finding is in accordance with the previous empirical research, mainly conducted in work settings [69,74,75,76,77], which supported that TWE can act as a catalyst for the predictive effect of TWC on IWB, strengthening it and fostering IWB. TB also moderated the relationship between TWC and IWB, in that at low levels of TB, IWB decreased as TWC was lower, whereas those college students with higher TWC developed more levels of IWB. At high levels of TB, the higher the TWC, the higher the levels of IWB exhibited by college students. This result is of particular interest. As we note, IWB comprises three distinct faces or forms of behavior, representing the three main stages of the innovation process [32], which are idea generation, idea promotion and idea realization. It could be suggested that these different processes and forms of behavior that make up IWB may require different levels of that emergent state of group interaction that is the TB to be effective [15]. Future research must explore such issues in HE from a longitudinal point of view.

### 5.1. Implications to Practice and Education

As we noted, the unique role of HE in contributing to innovation as well as to the development of other key competences, including teamwork, has been widely highlighted [41,42,43]. Our obtained set light around this issue. Incorporating active teaching and learning methodologies, such as GBL, could help HEIs to promote innovation and develop teamwork skills amongst college students in a healthy way, also allowing for the achievement of SDGs. One of the most consistent results around the effect of new active teaching and learning methodologies, such as GBL, in HEIs is their power to enhance engagement [49]. To foster IWB in HE, it is important to train teamwork-competent individuals, but also to create conditions that favor the experiences of TWE and TB while working in a group and GBL could be of great help to face such a challenge. There is a need, however, for further steps providing a clearer picture of which factors, especially those related to social interaction and teamwork, really matter regarding college students’ IWB in HEI.

### 5.2. Limitations and Suggestions for Further Research

This study is not exempt from limitations. First, our obtained results are based on a single data source—college students—and with a single collection method, a self-reported survey. Despite this, future research must use multisource and multimethod data (e.g., for example, observation, peer rating, etc.) to conduct new studies around the variables studied here; the so-called problem of common variance might be exaggerated [91,97]. Second, our study is cross-sectional in nature, so the results obtained do not allow causal relationships to be established. It is important that future research analyzes the findings from a longitudinal point of view. Third, the number of male students is lower than the number of female students frequently in Health Sciences and Social Work degrees, at least in the University of Zaragoza. Although our results did not show any effect of participant gender in the variables of our interest, it would be necessary to develop future studies that include a larger number of male participants. Fourth, as we noted, commonly, IWB is conceptualized and measured as a single dimension consisting of three different forms of behaviors, representing the three different stages of the IWB process. Some authors [98] suggested that this conceptualization of IWB might not properly capture its complex multidimensional character. As we noted, future research must explore if these three different forms of IWB might be related differently with TWC, TWE and TB in HEIs. Finally, our results were obtained in the context of a GBL experience in HE and may not be generalizable to other situations (e.g., expository class). It would be interesting to carry out future studies comparing different educational methodologies in HE.

## 6. Conclusions

Overall, our obtained results set light around fostering IWB in HE. Teamwork competence (TWC) constituted a key predictor for innovation. Those college students who are more competent in teamwork are better able to share and combine knowledge and ideas to a greater extent with their teammates and, therefore, they will be more innovative [27]. Teamwork engagement (TWE) and teambuilding (TB) moderated this effect. As we noted, innovation and teamwork have long been pointed out in the literature as two key competences for the 21st century [60,61] that should be developed and strengthened in HEIs. From a practical point of view, our results suggest that training college students in teamwork competences and cultivating TWE and TB while working in teams will favor their innovative capacity. Our findings could be added to those previous ones in contributing in HE to make it possible to establish more precise curricula guidelines to prepare innovative individuals capable of working in teams, contributing, in doing so, to the sustainability of innovation. One of the main limitations of our study is its cross-sectional nature. Future lines of innovation actions in HE may also encompass longitudinal research based on larger sample sizes.

## Figures and Tables

**Table 1:** Gender/degree distribution of participants.

	Occupational Therapy (*N* = 14)	Physiotherapy (*N* = 47)	Psychology (*N* = 54)	Social Work (*N* = 30)
Female (*N* = 103, 72.5%)	10	32	47	14
Male (*N* = 39, 27.5%)	2	14	7	16

**Table 2:** One-way ANOVA by group degree.

	Occupational Therapy (1)		Physiotherapy (2)		Psychology (3)		Social Work (4)			
	*N*	Mean (SD)	*N*	Mean (SD)	*N*	Mean (SD)	*N*	Mean (SD)	F	*p*
Innovation Work Behavior (IWB)	12	4.99 (0.94)	46	4.72 (0.78)	54	5.06 (0.78)	30	5.33 (1.03)	2.31	0.079
Teamwork Competence (TWC)	12	5.01 (0.60)	46	4.78 (0.54)	54	4.99 (0.90)	30	5.08 (0.51)	1.83	0.144
Teamwork Engagement (TWE)	12	5.08 (0.91)	46	5.03 (0.68)	54	5.93 (0.68)	30	5.37 (0.91)	1.89	0.145
Team Building (TB)	12	4.96 (0.73)	46	4.71 (0.53)	54	5.08 (0.68)	30	4.98 (0.81)	2.24	0.086

**Table 3:** Descriptive, reliability and correlational analyses.

Variable	M (SD)	α	1	2	3	4	5	6
1. Age	20.58 (1.61)	-						
2. Gender	-	-	−0.07	1				
3. Innovation Work Behaviors prior levels	4.52 (0.87)	0.92	0.68	−0.10	1			
4. Innovation Work Behaviors (IWB)	5.00 (1.02)	0.95	0.72	−0.14	0.62 **	1		
5. Teamwork Competence (TWC)	4.94 (0.59)	0.92	0.72	−0.15	0.48 **	0.72 **	1	
6. Teamwork Engagement (TWE)	5.25 (0.85)	0.95	0.10	−0.23	0.40 **	0.57 **	0.63 **	1
7. Team Building (TB)	4.93 (0.74)	0.93	0.60	−0.34	0.28 **	0.55 **	0.66 **	0.72 **

*Note. N* = 142. *M* and *SD* are used to represent mean and standard deviation, respectively. ** Indicates *p* < 0.01.

**Table 4:** Multiple additive moderation analysis of the explained variable IWB.

Variable	β	SE	t	*p*	95%	
					LLCI	ULCI
Age	0.00	0.03	−0.14	0.89	−0.07	0.06
Gender	−0.20	0.12	−1.70	0.09	−0.42	0.03
Innovation Work Behaviors prior levels	0.41	0.07	6.01	0.00	0.28	0.55
Teamwork Competence (TWC)	0.78	0.13	6.10	0.00	0.53	1.04
Teamwork Engagement (TWE)	0.17	0.09	1.80	0.07	−0.02	0.35
Team Building (TB)	−0.01	0.12	−0.07	0.95	−0.24	0.23
TWC × TWE	0.37	0.12	3.01	0.00	0.13	0.61
TWC × TB	−0.46	0.15	−3.03	0.00	−0.76	−0.16

*Note*. *N* = 142. β are unstandardized coefficients. R^2^ = 64%.

## Data Availability

The data presented in this study are available on reasonable request from the corresponding author. The data are not publicly available due to privacy restrictions.
